# CYFRA 21-1 is a prognostic determinant in non-small-cell lung cancer: results of a meta-analysis in 2063 patients

**DOI:** 10.1038/sj.bjc.6601851

**Published:** 2004-05-04

**Authors:** J-L Pujol, O Molinier, W Ebert, J-P Daurès, F Barlesi, G Buccheri, M Paesmans, E Quoix, D Moro-Sibilot, M Szturmowicz, J-M Bréchot, T Muley, J Grenier

**Affiliations:** 1 Montpellier Academic Hospital, France; 2 Thoraxklinik – Heidelberg gGmbH, Heidelberg, Germany; 3 Laboratoire de Biostatistiques, Institut Universitaire de Recherche Clinique, Montpellier, France; 4 Service d'Oncologie Respiratoire, Hôpital Sainte-Marguerite, Marseille, France; 5 Divisione di Pneumologia, Ospedale ‘S. Croce e Carle’, Cuneo I-12100, Italy; 6 Institut Jules Bordet, Brussels, Belgium; 7 Chest Disease Unit, University Hospital, Strasbourg, France; 8 Lung Cancer Research Group, CHU, 38043 Grenoble Cedex 9, France; 9 Department of Internal Medicine, Institute of Tuberculosis and Lung Diseases, Warsaw, Poland; 10 Hôtel-Dieu, Assistance Publique des Hôpitaux de Paris, 1 Place du Parvis Notre-Dame, 75181 Paris, France

**Keywords:** non-small-cell lung cancer, meta-analysis, CYFRA 21-1, prognosis

## Abstract

The purpose of this study was to determine the prognostic significance of a high pretreatment serum CYFRA 21-1 level (a cytokeratin 19 fragment) adjusted for the effects of well-known co-variables in non-small-cell lung cancer (NSCLC). This meta-analysis based on individual updated data gathered comprehensive databases from published or unpublished controlled studies dealing with the prognostic effect of serum CYFRA 21-1 level at presentation in NSCLC of any stage (nine institutions, 2063 patients). Multivariate regression was carried out with the Cox model. The proportional hazard assumption for each of the selected variables retained in the final model was originally checked by log minus log plots baseline hazard ratio. The follow-up ranged from 25 to 78 months. A total of 1616 events were recorded. In the multivariate analysis performed at the 1-year end point, a high pretreatment CYFRA 21-1 level was an unfavourable prognostic determinant in all centres except one (Hazard ratio (95% confidence interval): 1.88 (1.64–2.15), *P*<10^−4^). Other significant variables were stage of the disease, age and performance status. Within the first 18 months, the procedure disclosed a nearly similar hazard ratio for patients having a high pretreatment serum CYFRA 21-1 level (1.62 (1.42–1.86), *P*<10^−4^). For patients who did not undergo surgery, the hazard ratio during the first year of follow-up was 1.78 (1.54–2.07), *P*<10^−4^. Finally, in the surgically treated population, at the 2-year end point, a high pretreatment CYFRA 21-1 and a locally advanced stage remained unfavourable prognostic determinants. In conclusion CYFRA 21-1 might be regarded as a putative co-variable in analysing NSCLC outcome inasmuch as a high serum level is a significant determinant of poor prognosis whatever the planned treatment.

Treatment of non-small-cell lung cancer (NSCLC) is probably one of the great challenges of medical oncology owing to an increasing incidence in both men and women and poor prognosis (Ihde *et al*, 1997). Therapy of this disease remains experimental in many settings such as optimal chemotherapy combinations for the advanced stages. One of the difficulties in interpreting clinical trials and establishing treatment guidelines consists in the considerable heterogeneous clinical behaviour of this disease. Hitherto, the prognosis was mainly defined by three variables: the stage of the disease (Inoue *et al*, 1998; Andre *et al*, 2000), the performance status (Firat *et al*, 2002) and different patient conditions including age (Merrill *et al*, 1999). Although the negative impact of male gender remains debatable (Keller *et al*, 2002), gender is usually considered as an important variable and included in the stratification process of large randomised phase III trials (Schiller *et al*, 2002). This latter variable is not universally recognised as a prognostic factor, particularly in Europe where the female gender represents less than 30% of the population. Up till now, most of the randomised studies of chemotherapy in this disease are stratified on stage, performance status, and, inconstantly, presence of brain metastasis and weight loss (Scagliotti *et al*, 2002; Schiller *et al*, 2002). It is expected that such stratification avoids the imbalance of main prognostic variables between different treatment groups. However, the part of uncertainty remains high due to the great heterogeneity of tumour behaviour between groups defined according to the aforementioned variables. Thus, an awareness of significant variables able to predict poor prognosis is needed.

Several attempts at introducing biological variables into the ‘prognostic equation’ of NSCLC found different limits. Although some routine biological abnormalities such as elevated alkaline phosphatase, elevated lactate dehydrogenase, hyperleukocytosis or hyponatremia are well-known prognostic determinants in the setting of small-cell lung cancer (Cerny *et al*, 1987), their ability to predict patient outcome in NSCLC is inconsistently reported from one study to another (Paesmans *et al*, 1995b; Quoix and Moreau, 2000). On the other hand, sophisticated genetic abnormalities such as ploidy (Choma *et al*, 2001), p53 mutations (Steels *et al*, 2001), bcl-2 protein overexpression (Gaffney *et al*, 1994) need complex sample processes and techniques in order to detect a single abnormality.

Among the different biological markers that describe the NSCLC phenotype, one can consider the case of cytokeratin serum detection as a putative way to help in determining prognosis (Pujol *et al*, 1993a). As intermediate filaments of the epithelial lineage, cytokeratins indicate an epithelial differentiation. In addition, they might reflect the tumour growth fraction. Regarding the latter potential usefulness, tissue polypeptide antigen has been described as a human antigenic protein released immediately after mitosis (Björklund *et al*, 1987). A cytokeratin is a heterotypic tetramer of protofilaments composed of two polypeptides: one acidic type I subunit and one basic type II subunit. Each type of epithelia and their malignant counterpart express a specific cytokeratin polypeptide pattern (Moll *et al*, 1982, 1983). Simple epithelia, including pseudostratified epithelia such as the respiratory one, express cytokeratin 7, 8, 18 and 19. One of the most extensive experiences in this field is the use of tissue polypeptide antigen (TPA). Independent studies suggest that this marker is related to tumour mass and indicates a poor prognosis (Salvati *et al*, 1985; Buccheri and Ferrigno, 1989). However, immunological mapping has revealed that TPA contains 35 epitopes (Björklund *et al*, 1987). Selective antibodies raised against simple epithelium type cytokeratin, particularly the acidic (type I) subunit cytokeratin 19, have been shown to react with all histologies of lung cancers (Debus *et al*, 1984). A fragment of cytokeratin subunit 19 corresponding to epitope sequences lying within the sequences 311–335 and 346–367 (Bodenmüller *et al*, 1994) can be measured in serum by a sandwich assay, CYFRA 21-1, using two mouse monoclonal antibodies, KS 19-1 and BM 19–21.

CYFRA 21-1 has been extensively evaluated in the setting of NSCLC. This immunoradiometric test is well standardised and recognises a well-characterised cytokeratin 19 sequence. In addition, the sampling is not invasive and can be reproduced during follow-up. Interesting literature is emerging regarding the genetic regulation of cytokeratin 19 mRNA expression with differences from cell line to cell line (Ueda *et al*, 1999). Independent clinical groups have observed a similar negative prognostic effect of a high pretreatment serum CYFRA 21-1 level in NSCLC patient outcome.

By analysing the different prognostic studies (Ebert *et al*, 1993, 1997; Pujol *et al*, 1993a, 1996, 2001a; Giovanella *et al*, 1995; Moro *et al*, 1995; Paesmans *et al*, 1995a; Wieskopf *et al*, 1995; Szturmowicz *et al*, 1996; Brechot *et al*, 1997; Hamzaoui *et al*, 1997; Takei *et al*, 1997; Hirashima *et al*, 1998; Niklinski *et al*, 1998; Nisman *et al*, 1998, 1999; Foa *et al*, 1999; Kashiwabara *et al*, 2000; Buccheri *et al*, 2003), one can observe that there are some uncertainties regarding the exact hazard ratio of risk of death associated with a high serum CYFRA 21-1 level insofar as the estimated values range from 1.05 (Moro *et al*, 1995) to 2.8 (Wieskopf *et al*, 1995). This discrepancy is puzzling and might reflect both the relatively small size of the studies and the inconstancy of co-variables introduced in the proportional hazards model. For instance, the 1.41 hazard ratio reported in the recently updated Montpellier study (Pujol *et al*, 2001a) comprised a narrow 95% confidence interval (1.15–1.73) owing to the large population in which it was established. Therefore, we organised a meta-analysis of studies dealing with the estimation of the prognostic effect of CYFRA 21-1 in NSCLC. This meta-analysis aimed at defining, in over 2000 patients, the risk of death hazard ratio associated with a high serum CYFRA 21-1 level taking into account other main co-variables known as prognostic factors.

## TRIALS AND METHODS

### Eligibility criteria

This meta-analysis gathered complete and comprehensive databases from published or unpublished controlled studies dealing with the prognostic effect of pretreatment serum CYFRA 21-1 levels in NSCLC at any stage. Individual updated data have been used from all participating institutions.

To be accrued in the study each individual patient had to fulfil the following criteria: (1) histologically proven NSCLC according to the current World Health Organisation pathologic description of malignant tumours (World Health Organisation, 1999) in samples obtained either by bronchoscopy biopsies or fine needle trans-parietal biopsies or mediastinoscopy or any other sampling of metastases; (2) determination of stage grouping according to the following minimal staging procedure: clinical examination, standard chest roentgenography, computed tomographic (CT) scan of chest and upper abdomen (bone scanning and brain CT-scan were systematically applied in some centres only); (3) initial performance status (Zubrod *et al*, 1960) established according to the Eastern Cooperative Oncology Group (ECOG) scale (converted from Karnofsky index in one institution); (4) minimal description of the initial treatment that was dichotomously defined as surgery *versus* medical treatment (i.e. chemotherapy, radiotherapy, best supportive care or a combination of these therapies), and finally, (5) serum CYFRA 21-1 level as determined using either immunoradiometric assay or enzyme-linked immunosorbent assay prior to any therapy. Pooling data obtained via these two different techniques has been considered as methodologically valid insofar as the formal comparison of the results tightly correlated: 0.99 coefficient correlation after logarithmic transformation (Van Der Gaast *et al*, 1994).

### Selection of publications

A computerised bibliography was extracted from MEDLINE and CANCERLIT (CancerNet™) databases using medical subject headings for the following terms: lung neoplasm, lung carcinoma, non-small-cell, CYFRA 21-1, cytokeratin 19, and prognosis. The search for publications in any language was carried out from 1993, date of first prognostic study on CYFRA 21-1 (Pujol *et al*, 1993a), to 2001 inclusive. Afterwards, the manual selection of relevant studies was based upon summary analysis. The reprint of each study was carefully analysed regarding the different eligibility criteria. In addition to the aforementioned procedure, bibliographies of selected full papers were screened in order to disclose other relevant articles. Repeated publications regarding the same database were listed and the analysis was restricted to the most recent one (e.g. reference Pujol *et al*, 2001a for Pujol *et al*, 1993a, 1996, 2001a). The authors of each publication were invited to participate in this meta-analysis based on individual data. In addition to the literature search, some centres were directly contacted because they were renowned for working in the field of prognostic impact of cytokeratin markers in NSCLC. Most of them have presented their results as an abstract or a lecture during conferences but did not formally publish them until now. In addition, in 2001, the *Lung Cancer* journal published a letter by the Montpellier thoracic oncologic group advising that a meta-analysis based on individual data from all studies dealing with the estimation of CYFRA 21-1 as a prognostic determinant in this disease was ongoing and inviting all investigators interested in this project to participate (Pujol *et al*, 2001b).

The research procedure identified 16 putative centres that have communicated or published on the subject. Among the 16 centres contacted, 11 responded but only nine of them were able to produce a comprehensive database. Most of the studies have been published apart, some of them more than once. The total accrual of these centres represented a population of 2063 patients suffering from NSCLC (Table 1

**Table 1:** Database description

**Institution**	**Centre**	**Accrual**	**Median follow-up (months)**	**CYFRA 21-1 test**	**Reference**
Montpellier Acad. Hosp.	1	650	78	IRMA	Pujol *et al* (2001a)
Heidelberg Acad. Hosp.	9	439	56	ELISA	Ebert *et al* (1997)
Marseille Acad. Hosp.	2	291	50	IRMA	Hamzaoui *et al* (1997)
Cuneo Acad. Hosp.	7	180	27	IRMA	Buccheri *et al* (2003)
Jules Bordet Cancer Institute.	6	122	73	IRMA	Paesmans *et al* (1995)
Strasbourg Acad. Hosp.	3	116	25	IRMA	Wieskopf *et al* (1995)
Grenoble Acad. Hosp.	4	105	37	IRMA	Moro *et al* (1995)
Warsaw Acad. Hosp.	8	90	63	IRMA	Szturmowicz *et al* (1996)
Paris Acad. Hosp.	5	70	73	IRMA	Brechot *et al* (1997)

IRMA=immunoradiometric assay (Cis bio international, Gif/Yvette, France); ELISA=enzyme-linked immunosorbent assay (Roche formely Boerhiner Mannhein, Germany).

). Therefore, the population of the present meta-analysis consisted of 73% of the estimated accrual of all trials whatever the methodology and reliability of database. The estimated cumulative population, not included in the meta-analysis measured by using the reported patient numbers in the *methods* section of the publications, was 764 patients. This represents seven studies and eight publications (Giovanella *et al*, 1995; Takei *et al*, 1997; Hirashima *et al*, 1998; Niklinski *et al*, 1998; Nisman *et al*, 1998, 1999; Foa *et al*, 1999; Kashiwabara *et al*, 2000; Table 2

**Table 2:** Summary of studies not included in the meta-analysis

						**CYFRA 21-1 predictive prognostic significance**	
**Authors**	**Year**	**CYFRA 21-1 test**	**Accrual**	**Histology**	**Stage**	**Univariate**	**Multivariate**
Giovanella	1995	IRMA	148	All NSCLC	All	Significant	NA
Takei	1997	ELISA	87	All NSCLC	IIIB–IV	Significant	NA
Hirashima	1998	ELISA	149	All NSCLC	All	Significant	Independent
Niklinski	1998	IRMA	94	All NSCLC	Operable	Significant	Independent^a^
Nisman	1998 and 1999	ELISA	116	All NSCLC	All	Significant	Independent
Foa	1999	IRMA	62	All NSCLC	Operable	Significant	Independent
Kashiwabara	2000	ELISA	108	SQC	All	Significant	NA

NSCLC=non-small-cell lung cancer ; SQC=squamous cell carcinoma; NA=not available. Total accrual: 764.

a Adjusted and stratified log-rank test on TNM stage.

), all of them having reported a poor prognostic outcome for patients presenting with a high serum CYFRA 21-1 level, confirmed by multivariate analysis in four instances (Hirashima *et al*, 1998; Niklinski *et al*, 1998; Nisman *et al*, 1998, 1999; Foa *et al*, 1999).

### Collecting databases

On site help in preparing the data was provided by one author (OM) whenever this direct support was required. Each centre provided an updated database with the following variables: (1) centre number, (2) identification number of the patient in the centre, (3) gender, (4) age at time of diagnosis, (5) date of pretreatment CYFRA 21-1 sampling taken as the date of origin, (6) date of last contact, (7) status at last contact, (8) performance status, (9) staging carried out by standard procedures according to the 4th edition of the *Union Internationale Contre le Cancer* (UICC) tumour node metastases (TNM) classification (Tisi *et al*, 1982), the American Thoracic Society map of regional pulmonary nodes (Sobin *et al*, 1987) and the new Mountain stage grouping (Mountain, 1997), (10) histological subgroup (World Health Organisation, 1999), (11) pretreatment CYFRA 21-1 level, and (12) surgical resection or not. Histological classification was carried out according to five subgroups: adenocarcinoma, squamous cell carcinoma, bronchioloalveolar carcinoma, adenosquamous carcinoma and large cell carcinoma. Due to the low frequencies of both bronchioalveolar carcinoma and adenosquamous carcinoma (four and seven respectively), these histological subgroups were arbitrarily classified in the adenocarcinoma group. Therefore, only three histological modalities were analysed. Some centres prolonged their database accrual after their referred publications. The most updated accrual was taken into account.

### Statistical considerations

Survival was defined as the time from date of pretreatment serum CYFRA 21-1 sampling to the date of death. Death related to the disease whichever the progression site, or related to its treatment, was analysed as an event. Deaths from other causes were treated as censored observations. Survival distribution was estimated by the Kaplan and Meier (1958) method. Univariate survival analyses were carried out by means of log-rank tests.

Coding methods for the different variables depended on their nature. Some of the variables have been extensively described in the literature; therefore, the threshold has been defined from previous publications. Performance status has been analysed according to two classical modalities: PS 0-1 and PS greater than or equal to 2 (Zubrod *et al*, 1960). Stage has been coded according to the following three modalities: Ia–IIb, IIIa–IIIb and IV. Regarding the tumour marker (CYFRA 21-1), we used the first published threshold: 3.6 ng ml^−1^ (Pujol *et al*, 1993a). Histology has been coded according to the three aforementioned subgroups. Age was dichotomously tested as younger or older than the median age (63 years). The treatment modality was not tested as a prognostic variable inasmuch as treatment was decided according to each institution's procedure and was based upon the different pretreatment variables.

The date of origin of each database ranged from 23 February 1990 for the oldest to 2000 (seventh centre) for the newest. This long period led to great differences in median follow-up from one institution to another (Table 1), which precluded any definition of a unique time of survival update for all patients. Therefore, predefined time end points were used as fixed censored dates for repeated analyses. For the whole population time end points were 1 year and 18 months: for inoperable patients 1 year only, for operable patients 2 years only. In an attempt to determine significant variables during the first year of follow-up, participation of each individual patient in the survival probability was considered as true survival time for patients who died during this first year of follow-up. Otherwise, patients were right censored at 1 year. Similar calculations were attempted for the subsequent time end points (18 months and 2 years).

Multivariate regression was carried out with the Cox (1972) model. The classical forward selection of variable procedure was used. The selection of variables to be tested in the Cox model was made using the results of univariate analysis, that is,. variables reaching at least a p-level less than 15%. In addition, centre origin was considered as a variable in order to detect a centre effect. This model was written after a Boolean coding of the significant variables except for stage grouping that was left as a three-modality variable: categorical variables (such as performance status) were transformed into binary variables (0: negative or 1: positive). The number of levels of a Boolean variable needed to describe a predictive factor is one less than the categories of that factor inasmuch as its baseline level is defined by setting the value of each of the Boolean variables at zero. The significance of the effect of a given factor was assessed by determining whether or not the coefficient assigned to one or more of its categories was sufficiently different from zero. The proportional hazard assumption for each of the selected variables retained in the final model was originally checked by log minus log plots baseline hazard ratio. This procedure demonstrated the proportionality of hazard during the first 10-year period of follow-up and the first 18-month period of follow-up for the whole population, and during the first 2 years for patients who underwent surgery. For the subsequent time end points, the proportional hazard assumption was not verified which precluded Cox model analysis. The high number of events occurring during the first year of survival, and consequently the lower number of patients at risk following this landmark, was the explanation for the lack of proportionality. A *p*-level of less than 0.05 was considered significant. BMDP software package was used.

## RESULTS

### Patients' characteristics

In total, 2063 NSCLC patients were accrued by nine institutions (Table 1). Among them 75 (3.6%) were lost to follow-up. The median follow-up varied from 6 years 7 months for Montpellier Academic Hospital to 2 years 3 months for the Strasbourg Academic Hospital. A total of 1616 events was recorded during follow-up (78% of the patients). Characteristics of the global population are shown in Table 3

**Table 3:** Patients’ characteristics

**Variables**	
Median age (interquartile)	63 (54.9–69.0)
*Sex: number (rate)*	
Male	1740 (0.84)
Female	323 (0.16)
	
*Performance status (PS): number (rate)*	
PS_0_	378 (0.18)
PS_1_	682 (0.33)
PS_2_	474 (0.23)
PS_3_	197 (0.10)
PS_4_	57 (0.03)
PS not available	275 (0.13)
	
*Stage group: number (rate)*	
Ia–Ib	209 (0.102)
Iia–Iib	108 (0.052)
IIIa	287 (0.139)
IIIb	563 (0.273)
IV	886 (0.429)
Stage not done	10 (0.005)
	
*Histology: number (rate)*	
SQC	1039 (0.50)
ADE	721 (0.35)
LCC	303 (0.15)
	
*Induction treatment: number (rate)*	
Surgery	437 (0.21)
No surgery	1521 (0.74)
Not known	105 (0.05)
	
*CYFRA 21-1: number (rate)*	
⩽3.6 ng ml^−1^	1049 (0.51)
>3.6 ng ml^−1^	1014 (0.49)

SQC=squamous cell carcinoma; ADE=adenocarcinma; LCC=large cell carcinoma.

. The median age was 63 years and therefore chosen as a threshold in order to analyse survival according to this parameter. Female gender (16%) was underrepresented in comparison with the current sex ratio of NSCLC populations included in North American or recent European studies; a similar observation could be made for nonsquamous histologies (adenocarcinoma and large-cell carcinoma), which represented only one half of the population. Induction treatment (surgery *vs* chemotherapy or radio-chemotherapy) was fully recorded in all centres except one. A high serum CYFRA 21-1 level was detected in approximately one half of the whole population, a result in agreement with most of the individual studies.

### Whole population survival analysis (1 year and 18 months)

**Figure 1:**
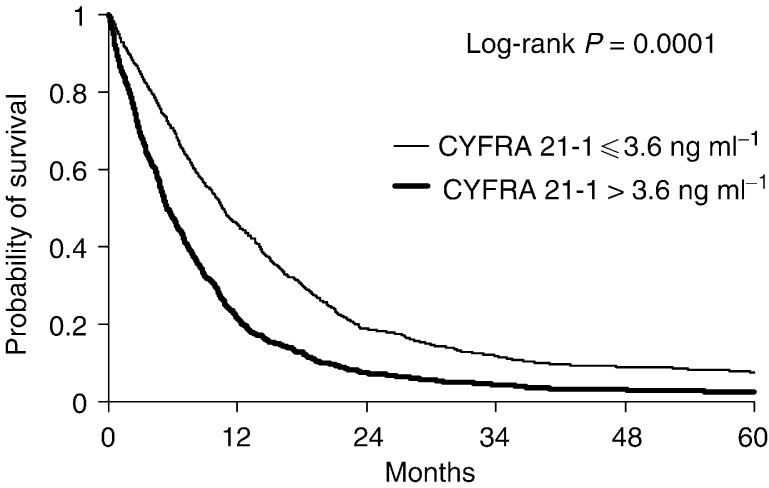
Probability of survival of non-small-cell lung cancer patients with normal and elevated pretreatment serum CYFRA 21-1 level; Kaplan–Meier curves were constructed taking into account the whole population survival.

Patients with a high serum CYFRA 21-1 level at time of presentation proved to have a shorter survival when compared with patients having a normal serum value (Figure 1

). Additional univariate analysis demonstrated a significant negative effect of the following features: an advanced stage, a performance status of 2 or higher, and age older than 63 years (*P*<10^−4^ for all log-rank tests). Histological subgroup and gender were not significant determinants of prognosis.

In the multivariate analysis performed at the 1 year end-point (Table 4

**Table 4:** Results of Cox proportional hazard model within the first year of follow-up for the whole population

	**Age**			**CYFRA 21-1**			**Stage grouping**			**PS**		
**Centre**	**HR**	**95 CI**	***P***	**HR**	**95 CI**	***P***	**HR**	**95 CI**	***P***	**HR**	**95 CI**	***P***
Montpellier	1.22	(0.99–1.50)	0.047	1.69	(1.37–2.09)	<10^−4^	1.59	(1.32–1.91)	<10^−4^	1.87	(1.52–2.31)	<10^−4^
Heidelberg	1.30	(0.87–1.94)	0.1	1.91	(1.26–2.90)	<10^−4^	1.34	(1.00–1.80)	0.05	1.44	(0.96–2.15)	0.07
Marseille	0.88	(0.61–1.27)	NS	2.22	(1.54–3.23)	<10^−4^	1.40	(1.00–1.94)	0.04	2.77	(1.74–4.42)	<10^−4^
Cuneo	0.79	(0.41–1.52)	NS	1.52	(0.84–2.74)	NS	1.22	(0.82–1.81)	NS	2.27	(1.25–4.14)	6 × 10^−3^
Bruxelles	1.56	(0.95–2.56)	0.07	1.93	(1.19–3.13)	6 × 10^−3^	1.63	(0.83–3.18)	NS	2.36	(1.25–4.43)	3 × 10^−3^
Strasbourg	1.11	(0.60–2.05)	NS	2.20	(1.07–4.52)	0.028	1.56	(0.89–2.76)	NS	3.23	(1.77–5.91)	<10^−4^
Grenoble	2.70	(1.56–4.67)	2 × 10^−4^	2.04	(1.18–3.52)	9 × 10^−3^	1.4	(1.00–1.97)	0.04	1.61	(0.93–2.81)	0.08
Warsaw	0.93	(0.53–1.65)	NS	1.72	(1.03–2.88)	0.034	1.72	(1.04–2.85)	0.03	1.38	(0.79–2.38)	NS
Paris	2.80	(0.97–7.92)	0.05	4.61	(1.64–12.7)	3 × 10^−3^	3.13	(1.46–6.69)	3 × 10^−3^	1.74	(0.61–4.96)	NS
												
Overall	1.26	(1.11–1.44)	4 × 10^−4^	1.88	(1.64–2.15)	<10^−4^	1.41	(1.28–1.56)	<10^−4^	2.01	(1.76–2.30)	<10^−4^

Codage of variables: all codages were binary except for stage grouping. Age: median age according to calculation in each centre. HR calculated in patients’ aged more than median age. CYFRA 21-1: HR affecting patients with a pretreatment serum CYFRA 21-1 level >3.6 ng ml^−1^. Performance status according to Zubrod ECOG system. HR affecting patients with a PS 2. Stage grouping according to the Mountain classification. This variable has been tested according to three modalities: Ia–IIb, IIIa–IIIb, and IV.

; Figure 2

**Figure 2:**
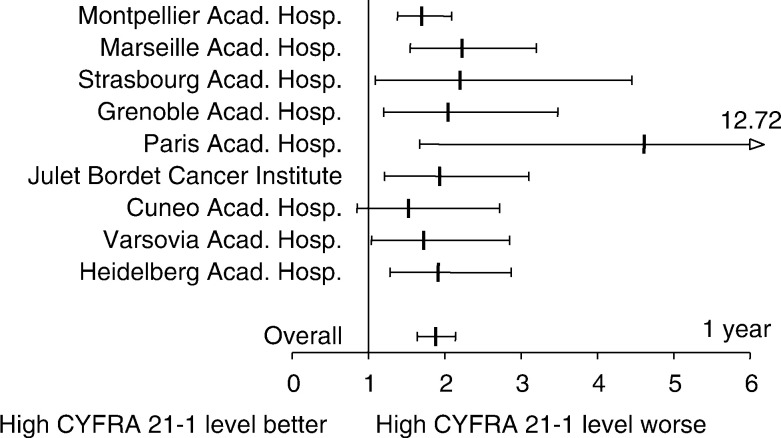
Hazard ratios and 95% confidence interval of mortality within the first year of follow-up for NSCLC patients who presented with a high pretreatment serum CYFRA 21-1 level. Results are expressed as individual and overall hazard ratios (vertical bars), and their respective 95% confidence intervals (horizontal bars). Hazard ratio higher than 1 indicates an increased risk of death for patients affected by a high serum CYFRA 21-1 level (*P*⩽10^−4^).

), CYFRA 21-1 was a prognostic determinant in each centre and reached statistical significance in eight out of nine centres. The overall hazard ratio (95% confidence interval) was 1.88 (1.64–2.15), *P*<10^−4^. Among the main classical prognostic factors, stage of the disease and performance status were both determinants of prognosis. In most of the centres both variables matched the criterion of statistical significance: performance status was a significant variable in five out of nine centres and stage grouping in six out of nine centres, one of them having a definite trend towards statistical significance (*P*=0.05). Therefore, PS and stage were less constant significant determinants when compared with the prognostic effect related to the high serum CYFRA 21-1 level. Although age was not a statistically significant prognostic determinant in a majority of centres, the overall evaluation demonstrated that an age older than 63 years indicated a poor prognosis (1.26 (1.11–1.44), *P*=4 × 10^−4^). Finally, there was a indubitable trend towards a significant centre effect as demonstrated by a hazard ratio of 1.02 (0.99–1.04) with a *P*-value of 0.05.

Within the first 18-month period, proportional hazard assumption was not verified for all variables in some centres. Overall, that precluded the definition of the hazard ratios for age and performance status. Nevertheless, the determination of hazard ratio of risk of death for patients having a high pretreatment CYFRA 21-1 level was possible inasmuch as the proportional hazard assumption was verified in all centres except one. Overall, the hazard ratio was in the range of the one calculated within the first year of follow-up: 1.62 (1.42–1.86), *P*<10^−4^ (Table 5

**Table 5:** Results of Cox proportional hazard model within the first 18-month follow-up period for the whole population

	**Age**			**CYFRA 21-1**			**Stage grouping**			**PS**		
**Centre**	**HR**	**95 CI**	***P***	**HR**	**95 CI**	***P***	**HR**	**95 CI**	***P***	**HR**	**95 CI**	***P***
Montpellier	1.20	(0.99–1.46)	0.053	1.48	(1.22–1.80)	<10^−4^	1.47	(1.25–1.74)	<10^−4^	‡	NA	
Heidelberg	0.96	(0.65–1.41)	NS	1.55	(1.04–2.32)	0.03	1.55	(1.14–2.10)	4 × 10^−3^	‡	NA	
Marseille	0.75	(0.52–1.08)	NS	2.04	(1.42–2.92)	<10^−4^	1.43	(1.09–1.89)	0.01	3.89	(2.38–6.34)	<10^−4^
Cuneo	0.82	(0.42–1.6)	NS	1.44	(0.79–2.60)	NS	1.24	(0.83–1.87)	NS	‡	NA	
Bruxelles	‡	NA		‡	NA		1.86	(0.98–3.47)	0.052	1.61	(0.9–2.85)	0.1
Strasbourg	‡	NA		1.92	(1.04–3.53)	0.03	1.65	(1.02–2.67)	0.04	2.78	(1.56–4.93)	4 × 10^−4^
Grenoble	1.98	(1.12–3.49)	0.016	2.21	(1.28–3.81)	4 × 10^−4^	1.42	(1.02–1.98)	0.04	1.45	(0.86–2.43)	0.16
Warsaw	0.86	(0.64–1.56)	NS	2.91	(1.67–5.08)	2 × 10^−4^	1.12	(0.681.85)	NS	2.18	(1.23–3.85)	6 × 10^−3^
Paris	2.27	(0.97–5.37)	0.055	3.63	(1.47–8.92)	4 × 10^−3^	4.16	(1.66–10.4)	3 × 10^−3^	1.17	(0.35–3.9)	0.79
Overall	‡	NA		1.62	(1.42–1.86)	<10^−4^	1.43	(1.30–1.56)	<10^−4^	‡	NA	

Codage of variables: All codages were binary except for stage grouping. Age: median age according to calculation in each centre. HR calculated in patients’ aged more than median age. CYFRA 21-1: HR affecting patients with a pretreatment serum CYFRA 21-1 level >3.6 ng ml^−1^. Performance status according to Zubrod ECOG system. HR affecting patients with a PS 2. Stage grouping according to the Mountain classification. This variable has been tested according to three modalities: Ia–IIb, IIIa–IIIb, and IV. ‡ Proportional hazards assumption not verified.

and Figure 3

**Figure 3:**
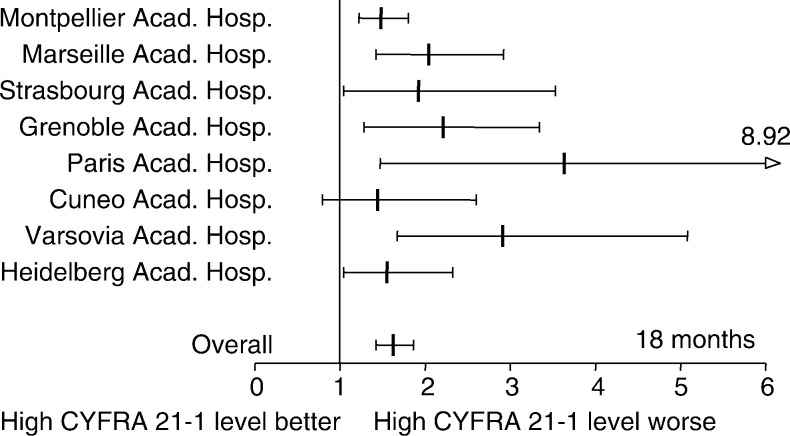
Hazard ratio and 95% confidence interval of mortality within the first 18-month follow-up period for NSCLC patients who presented with a high pretreatment serum CYFRA 21-1 level (symbols as in Figure 2; *P*⩽10^−4^). Results from the Jules Bordet Institute were not presented insofar as the proportional hazard assumption was not verified for these variables during the first 18-month period.

). Similarly, patients with an advanced stage were affected by a higher risk of death. No centre effect was disclosed in the proportional hazard model (HR=1.00 (0.98–1.03), *P*=0.63).

### Survival analysis at one year for patients who did not undergo surgery

The 1-year survival is considered as an important end point in clinical trials of chemotherapy in advanced NSCLC. Therefore, the Cox proportional hazard model was run again in patients who did not undergo surgery (*n*=1521). Proportional hazard assumption was matched in each centre for all variables that reached the statistical significance in univariate analysis (age, CYFRA 21-1, stage grouping and performance status). No centre effect was disclosed by the procedure: HR=1.02 (0.99–1.05), *P*=0.073. All four variables were independent determinants of prognosis: age: HR=1.15 (1.00–1.33), *P*=0.044; CYFRA 21-1: HR=1.78 (1.54–2.07), *P*<10^−4^; stage grouping: HR=1.33 (1.18–1.50), *P*<10^−4^; performance status: HR=2.01 (1.75–2.33), *P*<10^−4^.

### Survival analysis at 2 years for patients having undergone surgery

When the analysis was restricted to the 2-year survival rate in the surgically treated population the proportional assumption was verified for all variables and in all centres (*n*=437). No centre effect was disclosed (HR=1.04 (0.98–1.09), *P*=0.16). An advanced clinical stage (beyond stage IIb) remained the independent prognostic determinant of a poor survival (HR: 1.42 (1.1–1.83) *P*=5 × 10^−3^). A high pretreatment CYFRA 21-1 level also indicated a poor outcome although the hazard ratio did not reach the criterion of statistical significance, probably as a result of the smaller size of this subgroup. A definite trend towards statistical significance was observed, however (HR=1.41 (0.99–2.03), *P*=0.055).

## DISCUSSION

This meta-analysis aimed at accurately determining the hazard ratio of risk of death for NSCLC patients having a high pretreatment serum CYFRA 21-1 level compared to the group having a normal level. The Cox proportional hazard model was run at different predefined time end points, chosen according to their clinical significance. Considering the whole population, the overall hazard ratio for patients having a high pretreatment serum CYFRA 21-1 level was 1.88 (1.64–2.15) and 1.62 (1.42–1.86) during the first year and the first 18 month follow-up periods, respectively. For patients who did not undergo surgery, the hazard ratio within the first year was 1.78 (1.54–2.07) whereas for patients who underwent surgery the hazard ratio within the first 2 years was 1.41 (0.99–2.03).

The unfavourable prognostic significance of a high serum CYFRA 21-1 level was demonstrated at the early clinical development of this tumour marker. Following the first publications (Ebert *et al*, 1993; Pujol *et al*, 1993a), many confirmatory studies have been performed. Comprehensiveness is an important methodological issue when making a meta-analysis. It requires the amalgamation of all individual studies whatever the way of subsequent scientific communication (publication or meeting presentation). In the present meta-analysis, we made every effort to comply with the comprehensiveness requirement by individual contact with investigators and exhaustive publication research. Unfortunately, some databases were not eligible for meta-analysis; therefore, we present herein a population consisting of 73% of the estimated accrual of all trials whatever the methodology and reliability of databases. Contrasting with meta-analyses of clinical treatment trials, our meta-analysis belongs to a pattern of publications dealing with the prognostic significance of CYFRA 21-1. These prognostic studies are remarkable for their methodological heterogeneity. Differences in database reliability could be the result of the prospective or retrospective nature of the study. Important issues in this setting are organisation of the data collection, type of dosage, blindness of the biological measurements in order not to interact with clinical evaluation and treatment decision. Avoiding an important bias consists in taking treatment decision without knowledge of the studied variable. The nine databases that have been amalgamated herein comply with the best standards of prognostic study.

Due to the heterogeneity of follow-up duration from one study to another, a formal evaluation of survival needs the preplanned definition of time end points such as the 1-year survival rate in inoperable patients and the 2-year survival rate in patients who were operated upon. Consequently, the herein meta-analysis did not allow the determination of prognostic variables of long-term survival even though the median follow-up for the whole database was long enough. Significant variables have been determined considering participation of each individual patient. This method allows the amalgamation of databases, which differ according to the length of follow-up duration. The reliability of the statistical procedure could therefore be ascertained.

A limit of this meta-analysis consisted of the lack of homogeneity of staging procedure among the different centres. For this reason we considered that subgroup analyses according to stage grouping would have been unreliable. However, surgery was undergone in each centre according to recognised guidelines (Ihde *et al*, 1997). The same guidelines were used in proposing medical treatment to patients suffering from unresectable disease, particularly chemotherapy regimens. Best supportive care was proposed to patients with extremely poor performance status (13% of the overall population). We therefore dichotomised the meta-analysis into two subgroups: patients who underwent a complete surgical resection and patients who were not eligible for surgery and who received medical treatment only. The rationale for such a dichotomisation is based on day-to-day clinical practice. The prognostic significance of a high serum CYFRA 21-1 level was observed in both groups. In addition, multivariate analysis performed in the whole population demonstrated that both stage and serum CYFRA 21-1 level were independent prognostic determinants. Another point that could be discussed is the analysis of survival at 2 years for patients who underwent surgery. We were compelled to use this time end point in consideration of the major heterogeneity in follow-up duration from one centre to another. Finally, in the literature, the effect of age on NSCLC outcome has been evaluated using different cutoffs, the 70 year cutoff having been the most extensively used (Albain *et al*, 1991). Conflicting results have been reported. In our study we decided arbitrarily to use the median age (63 years) as the threshold. The effect of age on survival was observed in the whole population, but this result is restricted to our study and cannot be inferred to the general population of patients suffering from NSCLC.

The case of CYFRA 21-1, as a prognostic marker of NSCLC, might be discussed with regard to other putative tumour markers. A simple classification of tumour markers could be as follows: (i) oncofeotal markers and adhesion molecules: these markers are typical indicators of cancer phenotype (e.g. carcinoembryonic antigen); (ii) markers of cell lineage differentiation (CYFRA 21-1 belongs to this group of markers by detecting the epithelial lineage); (iii) markers of cell proliferation (e.g. thyrosine kinase); and finally, (iv) functional tumour markers. The last category includes important signalling pathways toward cell proliferation, cell differentiation, metastatic properties, antiapoptotic activity and angiogenesis. This field is in exponential growth as it is in close relationship with new targeted therapy approaches; adhesion molecules, extracellular-domain of epidermal growth factor receptor (ErbB 1) or Erb2 (HER2/neu), anti-p53 antibodies, belong to the functional tumour marker category.

Carcinoembrionic antigen (CEA) is a 180 kDa transmembrane glycoprotein belonging to the immunoglobulin superfamily. CEA is a complex of CEA itself, nonspecific cross-reacting antigen (NCA), and biliary glycoprotein 1. All genes belonging to this family are localised on chromosome 19 (Zimmermann *et al*, 1988). In this view, NCA constitutes the most prominent CEA immunoreactive molecule. In lung cancer cell lines, CEA is involved as a Ca^2+^-independent adhesion molecule in homotypic and heterotypic cell–cell binding. The CEA has been widely investigated as a serum tumour marker of many human malignancies, including lung cancer (Buccheri *et al*, 1987). Although its serum level is correlated with tumour stage in both small cell and NSCLC, its ability to help the prognostication and management of lung cancer is controversial.

Neural cell adhesion molecules (NCAM) are likely to be involved in the progression of lung cancer and, above all, in the phenotypic diversification of NSCLC. They are sialylated glycoproteins belonging to the immunoglobulin superfamily (Michalides *et al*, 1994). Their physiological role has been widely investigated and it is now well-recognised that NCAM are important molecules in the homotypic cell–cell relationship during the embryonic development of the brain. NCAM are composed of an intracellular domain and a transmembrane and extracellular domain. A single gene localised on 11q23 chromosome codes for all types of the NCAM family and alternative splicing of the large RNA segment result in different isoforms of NCAM which differ by their molecular weight (between 115 and 180 kDa). Above all, post-transcriptional modification of the molecule results in different NCAM characterised by the length of an *α*2,8 polysialic acid (PSA) chain linked-up to the extracellular domain of the molecule. This PSA branch is strikingly involved in the negative regulation of cell–cell adhesion. There is a concordant corpus of evidence that lung cancer is affected by a wide phenotypic heterogeneity. Non-small-cell lung cancer expresses the NCAM with a frequency of up to 20% of NSCLC specimens (Pujol *et al*, 1989).The heterotopic NCAM expression at the cell surface of NSCLCs has added a new observation to the list of evidence that the lung cancer phenotype of some tumours transgresses the frontier conveniently introduced between small cell and NSCLC. Several retrospective studies have been conducted in order to determine whether or not NCAM expression yields a more aggressive clinical behaviour (Berendsen *et al*, 1989) According to these studies, patients suffering from NSCLC with NCAM expression proved to have a shorter survival than those with a negative one. However, Cox model multivariate analysis revealed that nodal status and histology were the main independent determinants of prognosis (Pujol *et al*, 1993b).

p53 remains a major tumour suppressor gene inasmuch as its mutations result in an important step in lung cancer carcinogenesis (Miller *et al*, 1992). p53 mutations occur in all histological types at a frequency of 50–70%. There is a rationale to consider p53 as a putative prognostic factor of lung cancer: From a theoretical point of view, the p53 mutation resulting in an inactive protein leads to lack of control of cell proliferation by inhibiting the cell cycle arrest in the Gap 1 phase. Thus, tumour cells expressing this phenotype (abnormal p53 protein) are known to be genetically unstable (Kuerbitz *et al*, 1992). As this last feature is associated with cell diversification and tumour progression, it is tempting to analyse p53 mutation as a prognostic factor. Detection of anti-p53 antibodies in the serum has been proposed as a tumour marker for lung cancer. The rationale for this detection is based upon the observation that mutant p53 proteins accumulate and elicit immune reaction. In addition, the detection of serum anti-p53 Ab is highly specific to cancer. However, the test remains difficult to interpret and conflicting results have arisen from the literature (Murray *et al*, 2000; Zalcman *et al*, 2000).

Considerable attention is currently paid to the erbB family of receptor tyrosine kinases. There are four major identified receptors: HER1 (EFGR), HER2, HER3 and HER4. Ligands that interact with the extracellular domain are known for all members of the family except HER2. The latter, however, seems to play a key role in the process of heterodimerisation of the receptors, which in turn activates the intracellular tyrosine kinase domain towards cell proliferation, cell migration, angiogenesis, etc. HER-2/neu overexpression in tissue could be associated with cleavage of the extracellular domain. An ELISA method has been developed in order to detect the extracellular domain (monoclonal for capture; and polyclonal for detection). In a recent study, 84 patients with advanced NSCLC receiving CDDP-based regimen were analysed regarding the presence in the serum of the extracellular domain of HER-2/neu. According to the Cox model, the presence of this tyrosine kinase fragment is associated with a poor prognosis (Ardizzoni *et al*, 2001). Whether or not this information could be used in defining a population of patients candidate for anti HER-2/neu therapy remains to be defined.

Up till now, there is no single study able to simultaneously determine the respective prognostic significance of all aforementioned tumour markers. Therefore, it is not possible to establish whether or not the detection of serum cytokeratin 19 fragment (sequences 311–335 and 346–367) could be considered as better or equivalent to prognostic determinants of NSCLC when compared with the other new markers. Among the possible advantage of CYFRA 21-1 one can point out, the complete characterisation of the detected antigen, the knowledge of the function of cytokeratin, the well-established accuracy and reliability of the immunoradiometric assay and the large clinical evaluation of the marker in different clinical settings.

Most of the published or presented individual studies, including those that did not fit the methodological criterion for being included in the present study, reported a significant negative impact of a high serum CYFRA 21-1 level on NSCLC survival. The herein meta-analysis confirms this literature analysis and allows a precise estimation of the hazard ratio related to the marker. The congruence between the meta-analysis procedure and the individual reports strongly suggest that this is a true prognostic determinant.

Once having considered the reliability of the prognostic information and having measured the size of the effect on risk of death, the next step would be to determine how to integrate this information into therapeutic decision. Although of paramount importance, this question could not be answered in our study. Recently, the International Adjuvant Lung Trial (Le Chevalier, 2003) demonstrated that adjuvant chemotherapy after surgical resection of NSCLC induces a 4.1% absolute benefit at 5 years for overall survival. A putative field of applicability for pretreatment serum CYFRA 21-1 level titration would be to determine whether or not patients with a high level have a greater chance to benefit from this adjuvant therapy or whether there is need for a more aggressive multimodality treatment approach for these patients.

As of this moment, we conclude from our meta-analysis that CYFRA 21-1 might be regarded as a putative co-variable in analysing NSCLC outcome inasmuch as a high serum level is a significant determinant of poor prognosis whatever the planned treatment.
